# *ClPS1* gene-mediated manipulation of 2n pollen formation enables the creation of triploid seedless watermelon

**DOI:** 10.1186/s43897-025-00170-2

**Published:** 2025-09-02

**Authors:** Wenyu Pang, Qiaran Wang, Chenxin Li, Wenbing He, Jiafa Wang, Shujuan Tian, Li Yuan

**Affiliations:** State Key Laboratory of Crop Stress Resistance and High-Efficiency Production, College of Horticulture, Northwest A&F University, Yangling, Shaanxi 712100 China

**Keywords:** *ClPS1*, 2n pollen, Meiosis, Triploid watermelon

## Abstract

Seedless watermelons are increasingly dominating the consumer market due to their convenience and high quality. However, traditional triploid watermelon breeding faces challenges such as long breeding cycles and low survival rates of triploid F_1_ progeny, severely hindering both breeding and production. In this study, we identified the *ClPS1* gene as being associated with the formation of 2n gametes in watermelon. Expression analysis revealed that *ClPS1* is highly expressed during meiosis and microsporogenesis. Using CRISPR/Cas9, we generated *ClPS1*-targeted mutants, which disrupted chromosome segregation at metaphase II. This led to the production of diploid male spores and abnormal division of male spores, ultimately generating diploid pollen grains, while female meiosis remained unaffected. Moreover, self-fertilization or crosses using these mutants as paternal parents yielded triploid and aneuploid watermelons. Our findings demonstrate, for the first time, the molecular manipulation of 2n gametes to create triploid seedless watermelons, offering new insights into polyploid breeding and evolutionary studies in the *Cucurbitaceae* family and other species.

**Supplementary Information:**

The online version contains supplementary material available at 10.1186/s43897-025-00170-2.

## Core

Mutations in *ClPS1* cause parallel spindle formation during watermelon meiosis, producing unreduced or unevenly segregated gametes. The *clps1* mutant generated approximately 40% diploid microspores and pollen. Through selfing or hybridization, it successfully produced triploid seeds, demonstrating the first use of unreduced (2n) gametes for triploid seedless watermelon breeding. This simplifies traditional methods and provides a key theoretical basis for seedless fruit production in other crops.

## Gene and accession numbers

The sequence data for the watermelon *ClPS1* gene (ID: Cla97 C09G173120) was obtained from the Cucurbit Genomics Database (http://cucurbitgenomics.org/). Homologous protein sequences from other species were retrieved from the Arabidopsis Information Resource (TAIR, https://www.arabidopsis.org/), Phytozome (https://phytozome-next.jgi.doe.gov/), and the National Center for Biotechnology Information (NCBI, https://www.ncbi.nlm.nih.gov/).

## Introduction

Polyploids are widespread in the plant kingdom and exhibit a range of superior agronomic traits, making polyploid crops essential for agricultural production (Otto and Whitton 2000; Soltis et al. 2015). Sexual polyploidy, the primary pathway for polyploid formation in plants, has attracted increasing research attention. The discovery of unreduced (2n) gametes offers a novel perspective on the origin of polyploids (Otto 2007). Unreduced (2n) gametes are those that do not undergo the typical chromosome reduction during meiosis. These gametes are produced through various abnormal cytological mechanisms, including blocked cytoplasmic division, chromosome loss during meiosis, failed cell cycle transitions, and abnormal spindle orientations (De Storme and Geelen 2011; Peloquin et al. 1999). In addition, unreduced gametes can be categorized into two types based on their genetic composition: First Division Restitution (FDR) and Second Division Restitution (SDR). FDR-type 2n gametes contain the complete set of homologous chromosomes from the parent organism, thereby preserving and maximizing parental heterozygosity and epistatic interactions. In contrast, SDR-type 2n gametes consist of pairs of sister chromatids, leading to increased homozygosity and potentially reducing parental heterozygosity and epistatic effects (Cai and Xu 2007; Sun et al. 2021).

In recent decades, researchers have discovered some mutants that produce high-frequency unreduced (2n) gametes (Brownfield and Kohler 2010). For example, in maize, elongation (*el*) mutations have been found to cause the omission of the second division in female meiosis, resulting in the generation of FDR-type 2n female gametes (Barrell and Grossniklaus 2005). Potato mutants with premature cytokinesis or parallel spindle abnormalities during male meiosis have also been shown to produce 2n male gametes. Additionally, other potato mutants, such as the *fc* and *os* mutants, which exhibit female-specific failure of cytokinesis and omission of the second division, respectively, have been discovered to yield viable 2n egg cells (Peloquin et al. 1999). Although the phenotypes of these mutants have been reported, the related functional genes and their mechanisms of action remain unclear.

With the in-depth research, several genes crucial for the formation of unreduced (2n) gametes have been progressively identified (De Storme and Geelen 2013). In maize, mutations resulting from T-DNA insertion in the *ARGONAUTE104* and *AM1* genes, as well as in the *DYAD*/*SWITCH1* genes in *Arabidopsis*, disrupt homologous chromosome segregation, while sister chromatid division remains unaffected (Pawlowski et al. 2009; Ravi et al. 2008; Singh et al. 2011). This transformation of meiotic cells into mitotic-like cells ultimately leads to the formation of FDR-type 2n ovules with high heterozygosity. Similarly, disruption of cyclin complexes *OSD1*/*GIG1* and *TAM*/*CYCA2;1* in *Arabidopsis thaliana* causes abnormal sister chromatid separation during the second division, resulting in 2n pollen and egg cells with relatively high heterozygosity (Cromer et al. 2012; D'Erfurth et al. 2010, 2009; Iwata et al. 2011). Loss of the plant-specific gene *AtPS1*, responsible for parallel-spindle formation in *Arabidopsis*, leads to aberrant spindle structures, such as fusion spindles and tripolar spindles during meiotic metaphase II (Andreuzza and Siddiqi 2008; Brownfield et al. 2015; D'Erfurth et al. 2008). These spindle defects produce a large number of FDR-type 2n pollen, which represents the most common mechanism underlying 2n spore formation (Andreuzza and Siddiqi 2008). Another *Jason* mutant produces diploid SDR pollen grains via a male-specific meiotic restitution mechanism in *Arabidopsis* (De Storme and Geelen 2011).

Subsequently, researchers have demonstrated that the utilization of 2n gametes in plant breeding offers significant advantages for crop genetic improvement (Köhler et al. 2010; Younis et al. 2013). For instance, in potato, stress resistance from diploid species was successfully transferred to tetraploid cultivars using unreduced (2n) gametes (Capo et al. 2002; Carputo et al. 2000). Similarly, in alfalfa breeding, unreduced (2n) gametes have been employed to develop improved varieties with lower cyanide content and enhanced resistance to insect pests (Barcaccia et al. 2003). Meanwhile, unreduced (2n) gametes have also been proven to be feasible and useful in hybridization between plants of different ploidy levels, and can be used to construct sexual or meiotic polyploids (Consiglio et al. 2004; Khan et al. 2009). In the model plant *Arabidopsis thaliana*, the *OSD1* and *TAM*/*CYCA2;1* genes work synergistically to control the second division of meiosis. Single mutants or those producing diploid gametes cross with the wild type, resulting in triploid offspring (D'Erfurth et al. 2010, 2009). Additionally, mutations in the *SMC5/6* complex in *Arabidopsis* can also produce low-frequency male 2n gametes, leading to triploid offspring (Copsey et al. 2013; Yang et al. 2021). The *Jason* and *Atps1* genes in *Arabidopsis* are involved in controlling the orientation of the spindle during meiosis and specifically affect male gametes, with mutations resulting in triploid offspring (De Storme and Geelen 2011; D'Erfurth et al. 2008).

Watermelon is one of the most important economic fruits, and seedless watermelons are becoming increasingly popular due to their excellent flavor, good disease resistance, and favorable shipping qualities, thanks to their thick rind. Additionally, seedless watermelons tend to have a longer shelf life compared to standard watermelons (Wijesinghe et al. 2020). Traditionally, the breeding of triploid watermelons involves the artificial induction of tetraploid parental lines. This process typically requires treating seedlings with chemicals such as colchicine, which disrupt mitosis in somatic tissues, leading to tetraploid formation. These tetraploid plants subsequently produce 2n gametes (Sato et al. 2005). The tetraploid plants are then used as the female parent to cross with diploid male plants, resulting in the formation of triploid seeds. However, this method is labor-intensive, inefficient, and time-consuming. Additionally, polyploids generated through somatic doubling often exhibit greater variability, reduced fitness, and higher heterozygosity compared to those formed via sexual reproduction involving mutations that lead to unreduced (2n) gamete formation (Brownfield and Kohler 2010). Therefore, establishing a simple and effective molecular breeding method for triploid seedless watermelon will address many of the aforementioned issues. Based on the molecular mechanisms underlying the formation of unreduced (2n) gametes in *Arabidopsis* and the genetic background of the associated genes, strategies for inducing unreduced (2n) gametes in watermelon can now be developed. These strategies include the targeted knockdown of specific proteins to facilitate triploid watermelon breeding.

In this study, we focused on the role of the *ClPS1* gene in the formation process of unreduced gametes in watermelons. Through the analysis of *ClPS1* gene expression and function, we have highlighted its critical role in meiosis and have preliminarily elucidated the mechanism by which *ClPS1* mutations lead to the production of 2n pollen in watermelon. By self-crossing and crossing *clps1* mutants, we successfully obtained low-frequency triploid seedless watermelons, demonstrating for the first time the feasibility of hybridizing unreduced (2n) gametes in watermelon. The results of this study offer a novel perspective for triploid seedless watermelon breeding.

## Results

### *ClPS1* is highly expressed in meiosis and gametogenesis

**Fig. 1:**
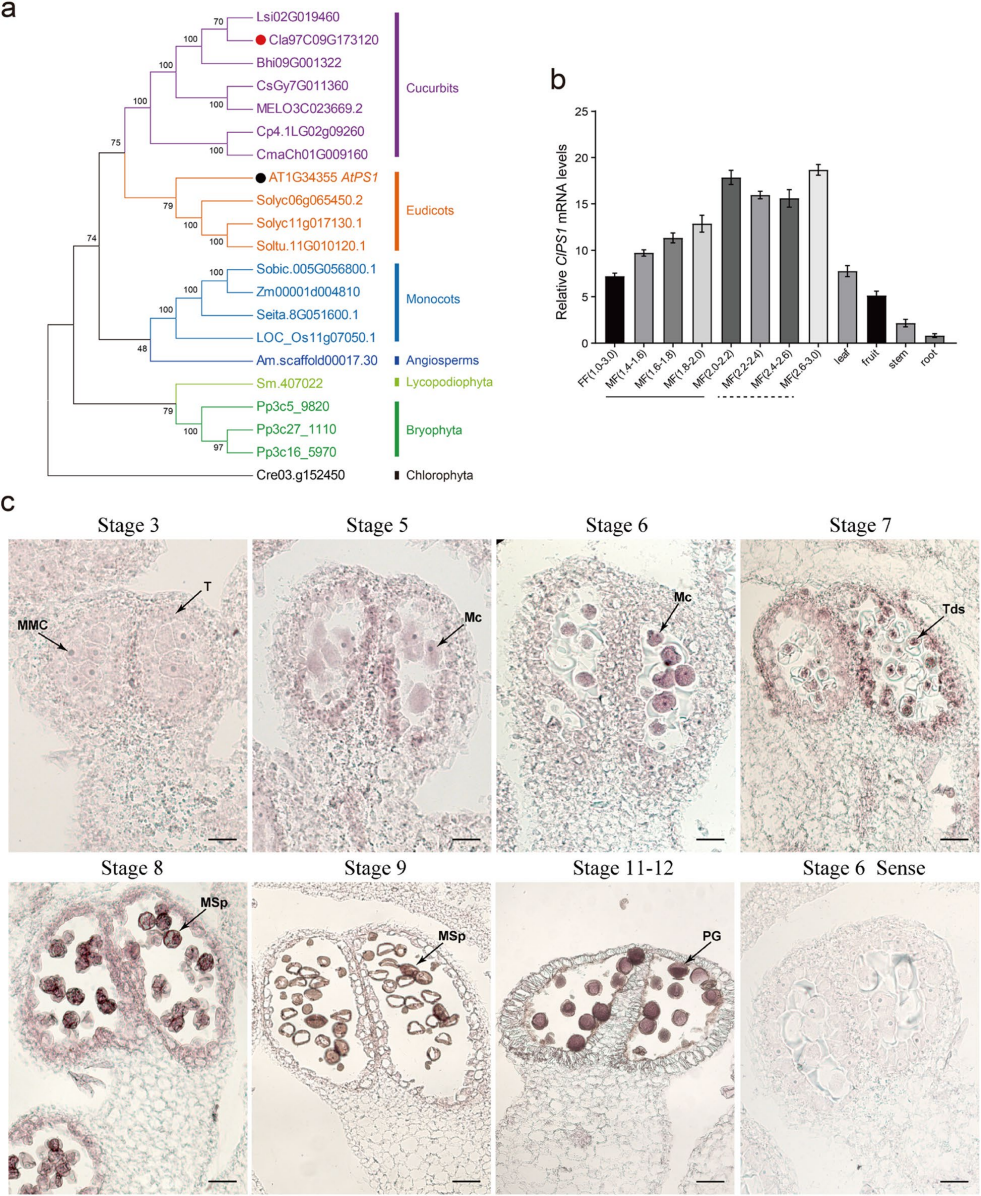
Identification of the *ClPS1* gene in watermelon. **a** Phylogenetic tree of PS1 predicted proteins from 18 plant species. Species codes: Am, *Amborella trichopoda*; At, *Arabidopsis thaliana*; Bh, Benincasa hispida; Cl, *Citrullus lanatus*; Cm, *Cucurbita maxima*; Cp, *Cucurbita pepo*; Cre, *Chlamydomonas reinhardtii*; Cs, *Cucumis sativus*; LOS_Os, *Oryza sativa*; Ls, *Lagenaria siceraria*; MELO, *Cucumis melo L*; Pp, *Physcomitrella patens*; Seita, *Setaria italica*; Sm, *Selaginella moellendorffii*; Sobic, *Sorghum bicolor*; Soltu, *Solanum tuberosum*; Solyc, *Solanum lycopersicum*; Zm, *Zea mays*. **b** Real-time quantitative analysis of the expression of *ClPS1* across various developmental stages in male flowers, female flowers, and vegetative tissues. Underlined and dashed lines indicate the meiotic phase and gametophytic stages, respectively. FF, female flower; MF, male flower. The numbers in brackets represent the flower diameter, measured in millimeters, classified and staged according to the watermelon reproductive calendar: MF (1.4–1.6), Microspore mother cells stage; MF (1.6–1.8), Early meiosis I; MF (1.8–2.0), Anaphase I; MF (2.0–2.2), Tetrad stage; MF (2.2–2.4), Microspore stage; MF (2.4–2.6), Uninuclear pollen stage; MF (2.6–3.0), Mature pollen stage. Error bars represent SE. **c** In situ hybridization analysis of *ClPS1* mRNA in different stages of watermelon male flower. T, tapetum; MMC, microspore mother cell; MC, Meiocyte; Tds, tetrads; MSp, microspore; PG, pollen grains. Scale bars, 50 μm

To identify the putative *PS1* gene in watermelon, we performed a BLAST search against the watermelon genome database (CuGenDB) using the known AtPS1 protein from *Arabidopsis thaliana* as a reference. This led to the identification of a candidate gene in watermelon, with the ID *Cla97 C09G173120*, which we designated as *ClPS1*. To investigate the evolutionary relationships of PS1 proteins across different species, we performed a phylogenetic analysis using sequences from 18 PS1 homologs from various species. The results revealed that, similarly to cucurbit crops including watermelon, almost all species had only one PS1 homolog, indicating a high level of conservation of the PS1 protein (Fig. 1a). Further protein sequence alignment (Figure S1) identified two conserved domains: the FHA domain at the N-terminal and the PIN domain at the C-terminal. Additionally, a SMART homology search revealed that homologs containing both FHA and PIN domains were not found outside the plant kingdom. The FHA domain is a phosphopeptide recognition motif that mediates protein–protein interactions and is involved in various cellular processes, including intracellular signaling, cell cycle regulation, transcription, DNA repair, and protein degradation. The PIN domain, on the other hand, is predicted to possess RNA-binding properties associated with RNase activity.

We investigated the expression pattern of *ClPS1* in watermelon using quantitative real-time polymerase chain reaction (qPCR) and in situ hybridization. Based on the meiotic reproductive calendar of watermelon (Jiang et al. 2024), male flower buds of varying diameters were collected and classified. These analyses revealed that a weak *ClPS1* transcriptional signal was first detected in microspore mother cells. As meiosis progressed, the transcriptional level of *ClPS1* mRNA increased significantly, reaching its peak at the tetrad stage. Furthermore, *ClPS1* mRNA remained relatively highly expressed during gametophyte development, with the highest expression observed in pollen grains. In contrast, the expression level of *ClPS1* in the roots and stems of watermelon was markedly lower (Fig. 1b,c). These results suggest that *ClPS1* is highly active during meiosis, particularly in meiosis II, and in gamete development.

### The *ClPS1* mutation leads to paternally inherited abnormal seed development

**Fig. 2:**
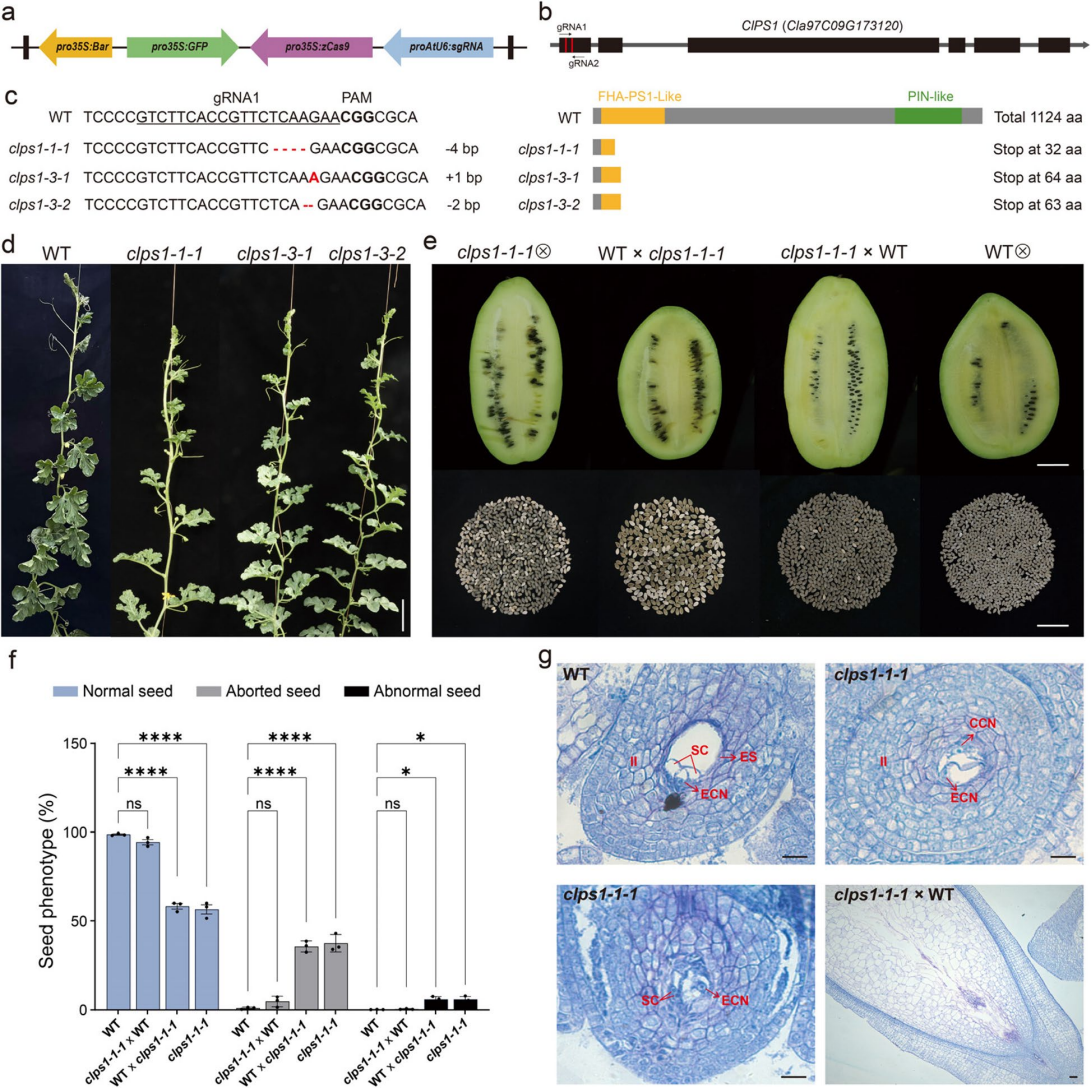
*clps1* mutants lead to paternally inherited abnormal seed development. **a** The schematic diagram of the CRISPR/Cas9 gene-editing vector used in this study. **b** Schematic diagram of the *ClPS1* gene. **c.** Target sequences and actual editing results in different *clps1* mutant plants. The PAM sequence is displayed in bold font, and the target sequence of 20 bp is marked with a line. Insertions or deletions are indicated in red. **d** WT and the *clps1* mutant plants. Scale bars, 10 cm. **e** Seed production in WT, *clps1-1–1* mutant and their reciprocal crosses. The white empty shell is aborted seeds. Scale bars, 5 cm. **f** Percentage of normal seeds and aborted seeds in manually pollinated WT, *clps1-1–1* mutant, and their reciprocal crosses. Significance in Fisher’s exact test, *P < 0.005, **P < 0.00001. **g** The Semi-thin paraffin sections of WT, *clps1-1–1* ovule on the day of flowering and the cross progeny (*clps1-1–1* × WT) ovules 8 days after pollination. II, inner integument; SC, synergid cell; ES, embryo sac; ECN, egg cell nucleus; CCN, central cell nucleus. Scale bars, 20 μm

To further investigate the role of *ClPS1* in watermelon, we used CRISPR/Cas9-based gene editing to target two sequences within the first exon of the watermelon variety ‘YL’ (Fig. 2a,b). A total of 7 *T*_*0*_ transgenic lines were obtained, and Sanger sequencing confirmed varying degrees of mutations at the target sites in all these lines (Table S1). Among them, *clps1-1*, *clps1-4*, and *clps1-7* were heterozygous edited plants, while the others were homozygous or biallelic edited plants. To exclude off-target effects, we predicted potential off-target sites using CRISPR-P v2.0 (http://crispr.hzau.edu.cn/cgi-bin/CRISPR2/CRISPR). Sequencing results indicated no mutations at the predicted off-target sites (Table S2).

To obtain homozygous mutants free of the Cas9 gene, we screened 16 *T*_*1*_ plants using GFP fluorescence and PCR identification. Three mutant lines (*clps1-1–1*, *clps1-3–1*, *clps1-3–2*) without Cas9 were successfully acquired. At the first target site, deletions of 4 bp and 2 bp, as well as an insertion of 1 bp, resulted in the truncation of the ClPS1 protein and the loss of conserved domains (Fig. 2c). Notably, both homozygous and biallelic mutants exhibited identical phenotypic characteristics. Therefore, *clps1-1–1* was selected as the representative line for phenotypic analysis.

The *clps1* homozygous mutant did not exhibit significant abnormalities in vegetative growth, fruit development, or ripening compared to the wild-type (WT) strain (Fig. 2d). However, when self-crossed, the *clps1 - 1–1* mutant exhibited a seed abortion rate of 37.5%, compared to just 0.40% in self-crossed WT plants (Fig. 2e, f). Additionally, we observed 5.94% of abnormal seeds, which showed wrinkles or even deformities in the offspring (Fig. 2f, Figure S2). To determine whether seed abortion was due to male or female developmental defects, we conducted a reciprocal cross experiment between the *clps1 - 1–1* mutant and WT plants. When the *clps1 - 1–1* mutant was used as the male parent, a seed abortion rate of 35.8% and an abnormal seed rate of 5.98% were observed. However, when the *clps1-1–1* mutant was used as the female parent and pollinated with WT pollen, no significant seed abortion was observed (Fig. 2e,f). This suggests that the high rate of seed abortion in the mutant was caused by defects in the male parent.

To verify this conclusion, we performed semi-thin paraffin sectioning of mature *clps1-1–1* embryo sacs and ovules pollinated with WT pollen. The structure of the embryo sac remained intact compared to that of wild-type mature embryo sacs, and no abnormalities were observed in the development of egg cells, central cells, or endosperm nuclei. Additionally, no abnormalities were detected in embryo development after 8 days of pollination with WT pollen (Fig. 2g). These results indicate that the *clps1-1–1* mutation does not cause aberrant development in female gametophytes or embryos. In summary, disruption of *ClPS1* leads to abnormal seed development in watermelon, with the defect being paternally inherited.

### Mutations in the *ClPS1* lead to diploid spores and pollen

**Fig. 3:**
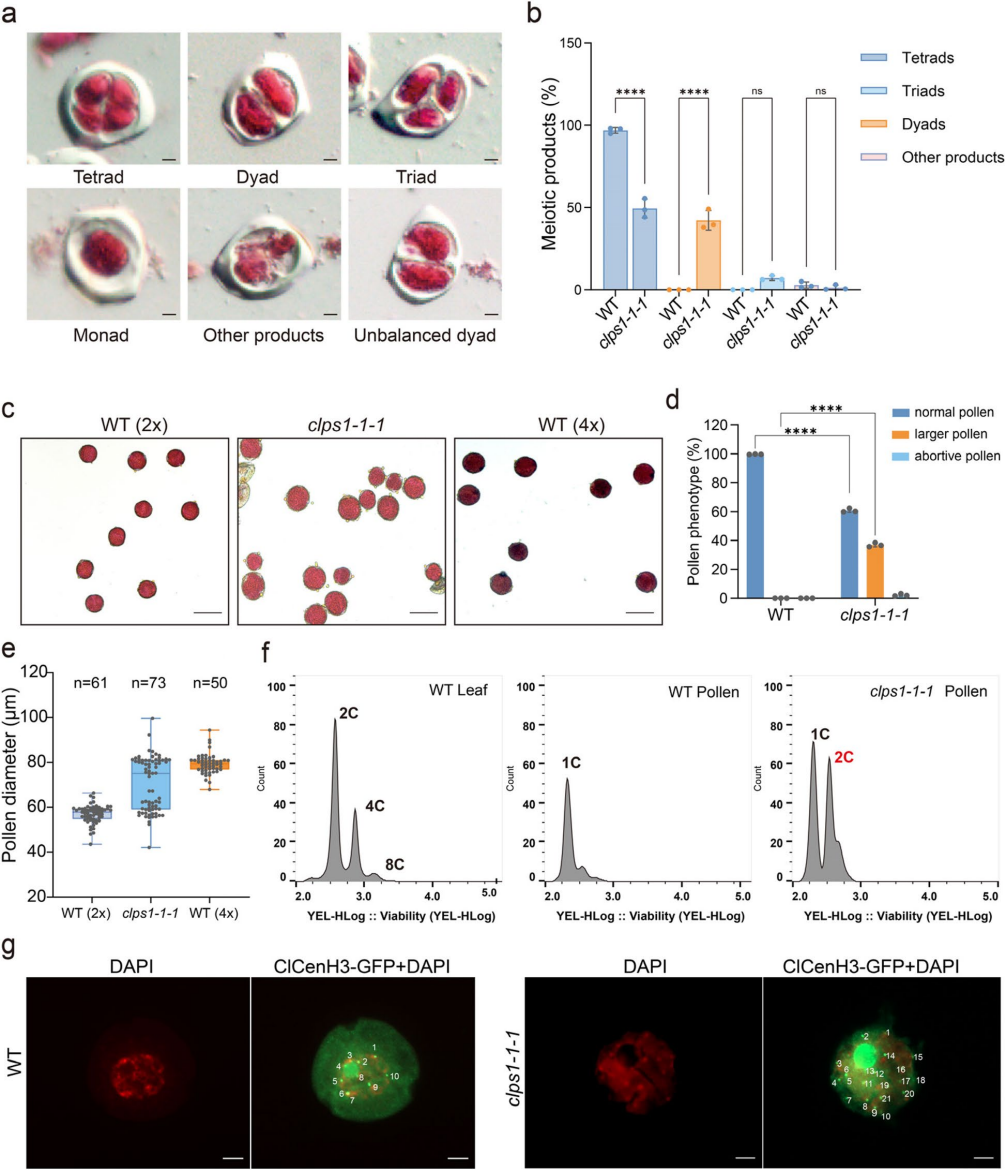
*clps1* mutants produce normal and 2n pollen. **a** Meiotic products and activity analysis in *clps1* mutants. Scale bar, 20 μm. **b** The percentage of different meiotic products (dyad, triad, tetrad, and others) in WT (*n* = 95) and *clps1-1-1* (*n* = 89). **c** Analysis of pollen viability by Alexander staining in mature pollen of 2 × WT, *clps1-1-1* and 4 × WT plants**.** Note uniform sizes of pollen grains produced by WT and different sizes of pollen from *clps1-1-1* mutant plants. And the larger pollen appears to be the same size as that of tetraploid plants. Scale bars, 100 μm. **d** Percentage of normal pollens, larger pollens, and aborted pollens in WT and *clps1-1-1* mutant. Significance in Fisher’s exact test, *P < 0.005, ****P < 0.00001. **e** Box plot showing the diameter of individual pollen grains in diploid (2 × WT) and tetraploid (4 × WT) and *clps1* plants. The numbers above violins indicate the mean diameter. **f** Flow cytometric analysis of wild type and *clps1-1-1* mutant pollen nuclei. **g** Immunofluorescence of WT and *clps1-1-1* mutant pollen. ClCenH3 centromeric protein was used as the first antibody and stained with DAPI (red). Scale bars, 5 μm

To further investigate the causes of seed abortion in the *clps1* mutant, we assessed the viability and fertility of meiotic products in the *clps1-1-1* mutant. In wild-type (WT) plants, male meiosis generates a group of four spores arranged in a tetrahedral formation, known as a tetrad. We isolated anthers at the tetrad stage from male flowers and performed Alexander staining. The results revealed that, in addition to normal tetrads, the *clps1* mutant produced viable dyads, triads, and other abnormal meiotic products (Fig. 3a). Among these, dyads accounted for 42.3% (Fig. 3a,b).

To assess the viability of pollen grains, we performed Alexander staining on pollen from the *clps1-1–1* mutant. The results indicated that both dyads and triads produced viable pollen grains. However, we observed a surprising variability in the size of *clps1-1-1* pollen grains, which sharply contrasts with the uniform size of WT pollen (Fig. 3c). Statistical analysis revealed that 36.9% (*n* = 92) of the pollen grains were larger than normal (Fig. 3d). Upon measuring the diameters of the pollen grains, we found that in WT plants, the average pollen diameter was 57.02 ± 4.01 μm (*n* = 61). In contrast, *clps1-1–1* mutant pollen grains exhibited significant size variation, with the population roughly divided into two groups: one with diameters ranging from 40.2 to 63.9 μm, and the other from 73.6 to 99.6 μm (Fig. 3e). The average diameters of these two groups were 57.83 ± 4.18 μm (*n* = 35) and 80.29 ± 4.54 μm (*n* = 38), respectively (Fig. 3e). Thus, the *clps1-1-1* mutant produces two distinct cohorts of differently sized pollen.

Notably, pollen size exhibited a positive correlation with its nuclear DNA content, and the size increased proportionally with the nuclear DNA content. We postulated that the larger pollen grains observed in *clps1-1-1* mutant were not haploid. To validate this hypothesis, we measured the diameters of tetraploid watermelon pollen. The results showed that the average diameter was 79.34 ± 4.42 μm (n = 50), which closely matched the larger pollen grains observed in the *clps1-1-1* mutant (Fig. 3e).

To directly confirm the diploidy of the larger pollen in the *clps1* mutants, we used flow cytometry to determine the ploidy levels of the pollen. Mature pollen was collected from both WT and *clps1-1–1* plants, with WT leaves serving as a control. The results showed that almost all WT pollen contained only 1C nuclei (Fig. 3f). In contrast, *clps1-1–1* pollen displayed not only the typical 1C signals but also additional signals consistent with 2 C nuclei, which were also observed in the leaf cells of WT plants (Fig. 3f). This strongly suggests that the *clps1-1–1* mutant produces both haploid and diploid pollen grains. To further confirm this, we performed immunofluorescence analysis on *clps1 - 1–1* pollen using an antibody targeting the watermelon centromeric protein ClCenH3 (Fig. 3g). The results revealed that the larger pollen grains in the mutant contained approximately twice the chromosome number of the wild type. Together, these findings provide compelling evidence that the *clps1* mutant produces diploid pollen grains.

### Parallel spindle formation in the *clps1* mutant led to the unreduced 2n male gametes

**Fig. 4:**
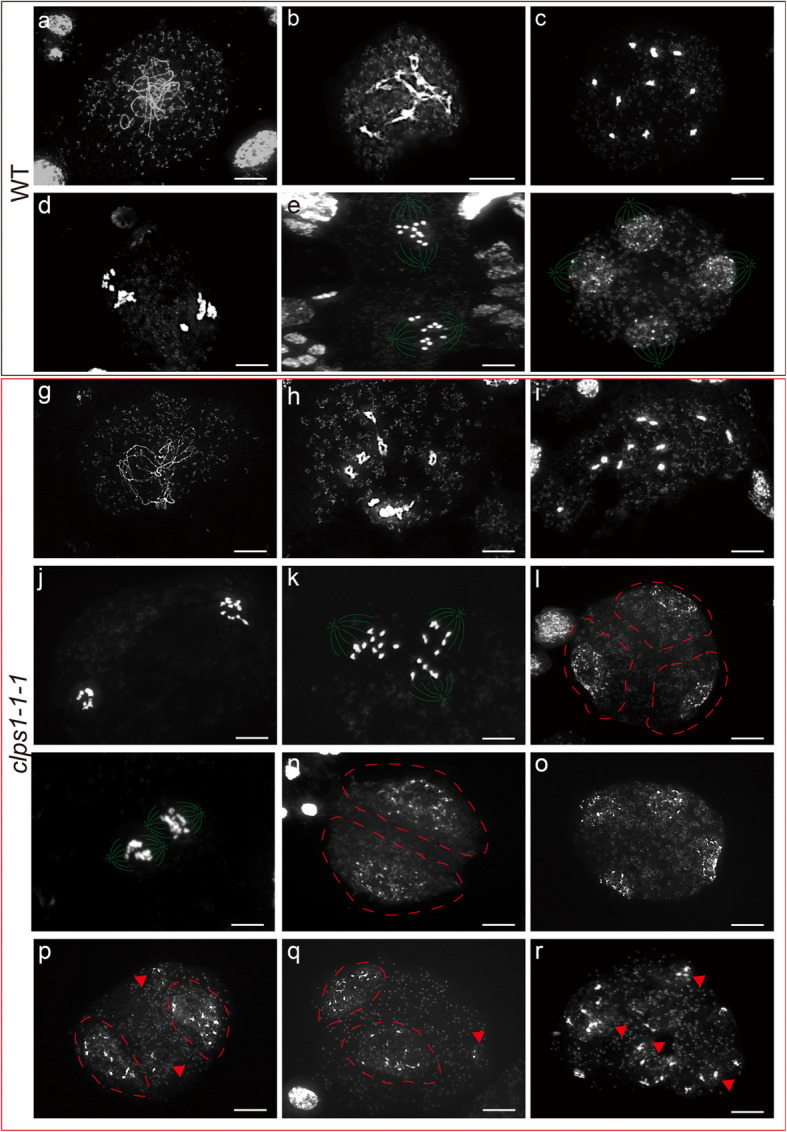
Chromosome segregation defects during meiosis II in *clps1* mutants. **a–f** Wild type meiosis chromosome spreads. **a** Prophase I pachytene. **b** Prophase 1 diplotene. **c** Metaphase I. **d** Anaphase I. **e** Metaphase II. **f** Telophase II. **g–r** *clps1-1-1* meiosis chromosome spreads. **g** Prophase I pachytene. **h** Prophase I diplotene. **i** Metaphase I. **j** Anaphase I. **k** and **m** Metaphase II. **l** Triad. **n** Dyad. **o** tetrad. **p** Unbalanced tetrad. **q** Unbalanced triad. **r** Polyad. Use green lines to simulate the spindle. Scale bar, 10 μm

To uncover the mechanism behind the production of dyads in the *clps1-1-1* mutant, we analyzed chromosomal behavior during meiosis. Chromosome spreads revealed that meiosis I in the *clps1-1-1* mutant proceeded normally, similar to wild-type plants (Fig. 4). Homologous chromosome synapsis was completed in prophase I, and chiasmata formed bivalents, which manifested as crossovers (Fig. 4b,h). Chromosomes moved to the poles of the cells during anaphase I (Fig. 4d,j).

However, metaphase II exhibited distinct differences in the *clps1-1–1* mutant compared to wild-type plants. In the wild type, chromosomes were arranged on two separate metaphase II plates, each containing 11 chromosomes (Fig. 4e). In contrast, some *clps1-1–1* cells showed abnormal chromosome arrangements, with 22 chromosomes distributed in the same plane, with no tendency to separate (Fig. 4k). At telophase II, wild-type cells divided equally to form tetrads (Fig. 4f), while in the *clps1-1–1* mutant, in addition to normal tetrads, we observed the formation of dyads, triads, and unequal division of spores (Fig. 4l-r). These observations are consistent with previous findings that the meiotic products in the *clps1* mutant are a mixture of dyads, triads, and tetrads.

In most dicotyledonous plants, male meiosis progresses with simultaneous division, resulting in two spindles within a single cell during meiosis II. The integrity of the spindles is crucial for proper chromosome alignment and separation. In wild-type plants, the metaphase II spindles are typically arranged perpendicular to each other (Fig. 4e), leading to the formation of four well-separated poles and tetrads during anaphase II (Fig. 4f). In contrast, in the *clps1-1–1* mutant, all 22 chromosomes that are morphologically intact during metaphase II (Fig. 4k, m) indicate that the spindle shape remains regular. However, the chromosomes appear aggregated instead of being separated into two distinct groups, suggesting that the directions of the two spindles attached to the chromosome centromeres have shifted. Instead of being perpendicular as in the wild type, the spindles are now parallel (Fig. 4m) or even tripolar (Fig. 4k). This spindle misorientation explains the formation of triads and dyads. These abnormal spindle configurations cause chromatids that had separated during meiosis I to aggregate again during anaphase II. Additionally, occasionally, three to four groups of chromosomes surrounded by spindles are dispersed within the cell (Fig. 4p-r). In conclusion, this spindle defect likely accounts for the observed unbalanced meiotic products in the *clps1* mutant.

### Loss of *ClPS1* results in aneuploid offspring

**Fig. 5:**
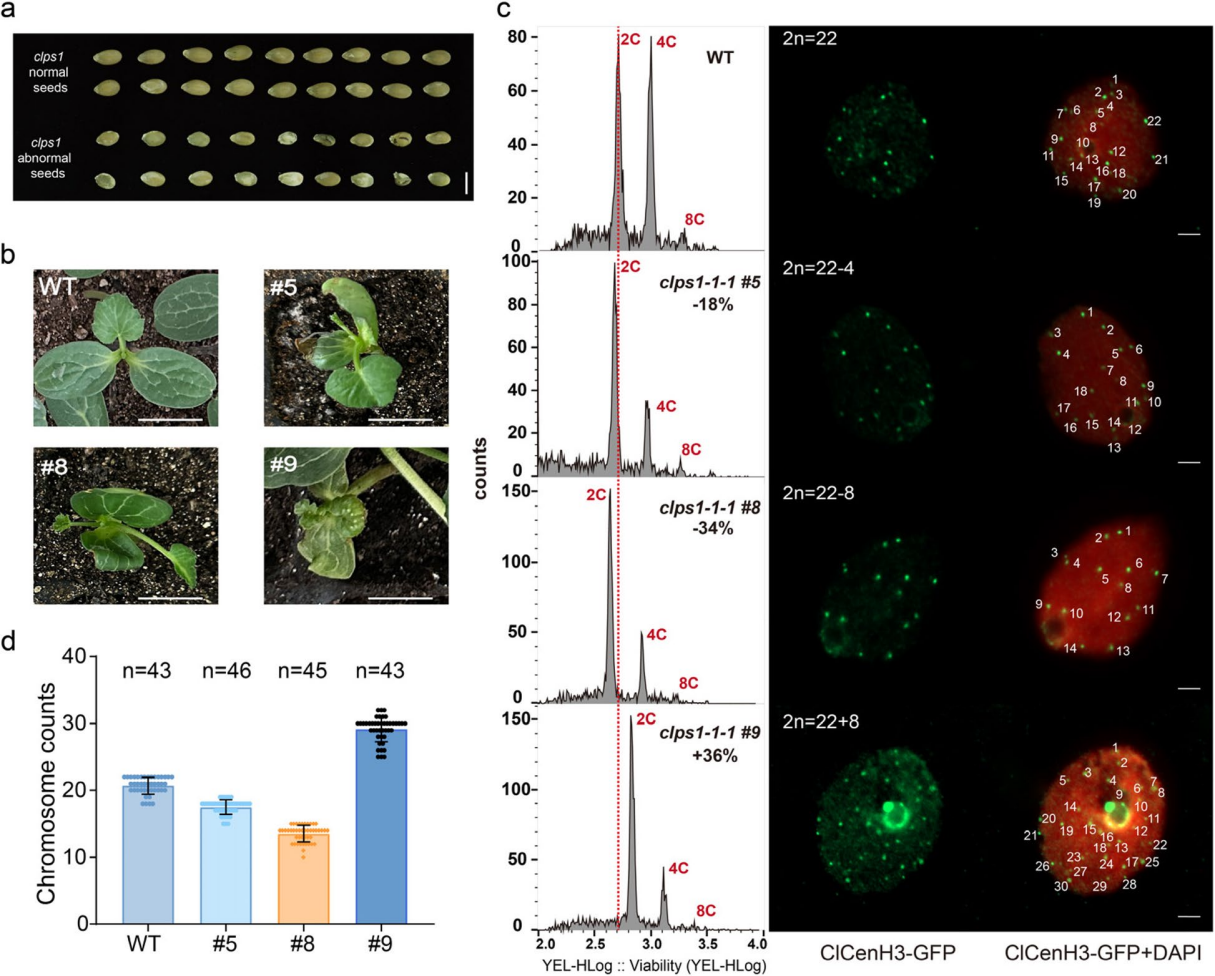
Loss of the *ClPS1* results in aneuploidic offspring. **a** Comparison of *clps1* mutant abnormal seeds and wild type seeds. Scale bars, 1 cm. **b** Examples of seedling abnormalities in *clps1* progeny. Individuals #5, #8, and #9 represent distinct aneuploid watermelon specimens. Scale bars, 2 cm. **c** Flow cytometric analysis and chromosome count of *clps1* abnormal seed progeny. Left, Relative DNA content of ‘YL’ wild-type and four individual *clps1* offspring plantlets with different amounts of surplus DNA measured by flow cytometry. Right, Immuno-staining of *clps1* offspring plantlets nuclei using a watermelon centromere-specific CenH3 antibody. DNA was counterstained with DAPI (in red). Centromere signals (in green) indicate the chromosome number. Scale bars, 2 μm. **d** Chromosome count statistics of wild-type and aneuploidic offspring in immunofluorescence assay

The *clps1* mutant produces viable unequal divisions, resulting in dyads, triads, and other abnormalities, which we speculate could lead to aneuploid progeny. Notably, the proportion of abnormal seeds is significantly higher in the mutant compared to the wild type. We sowed abnormal seeds from the *clps1-1–1* mutant and observed the resulting seedlings. We found that many of the seedlings from these abnormal seeds exhibited morphological abnormalities, deformities, or even ceased growth (Fig. 5a,b and Figure S3). To further determine whether the deletion of *ClPS1* directly affects the surviving offspring, we measured the relative DNA content of progeny from the *clps1* mutant's abnormal seeds using flow cytometry, with wild-type plants serving as the internal control for comparison. The results showed that the 2 C DNA peaks of plants #5 and #8 were shifted by − 18% and − 35%, respectively, compared to the wild-type control group, indicating that the DNA content in these plants was lower than in wild-type plants. In contrast, the #9 plant showed higher DNA content compared to the wild type (Fig. 5c). These results suggest that plants #5, #8, and #9 exhibit aneuploidy due to abnormal chromosome numbers.

To confirm the flow cytometry data, we performed an immunofluorescence assay using a watermelon centromere-specific antibody, ClCenH3, on the 2 C leaf nuclei of *clps1* plants with varying DNA content. The maximum number of fluorescent signals corresponded to the number of centromeres, thereby indicating the chromosome count in individual mutants (Fig. 5c,d). In plants with 18%, 35%, and 40% differences in DNA content from the wild type, we observed an average of 17.5 ± 1.0 (*n* = 46), 13.6 ± 1.1 (*n* = 45), and 29.1 ± 1.9 (*n* = 43) fluorescent signal spots, respectively. In comparison, wild-type plants showed 20.7 ± 1.3 signals (Fig. 5d).

These findings clearly demonstrate that the *clps1* mutant plants produce aneuploid microspores, which participate in fertilization and lead to the generation of aneuploid offspring.

### *clps1* mutants produce triploid offspring

**Fig. 6:**
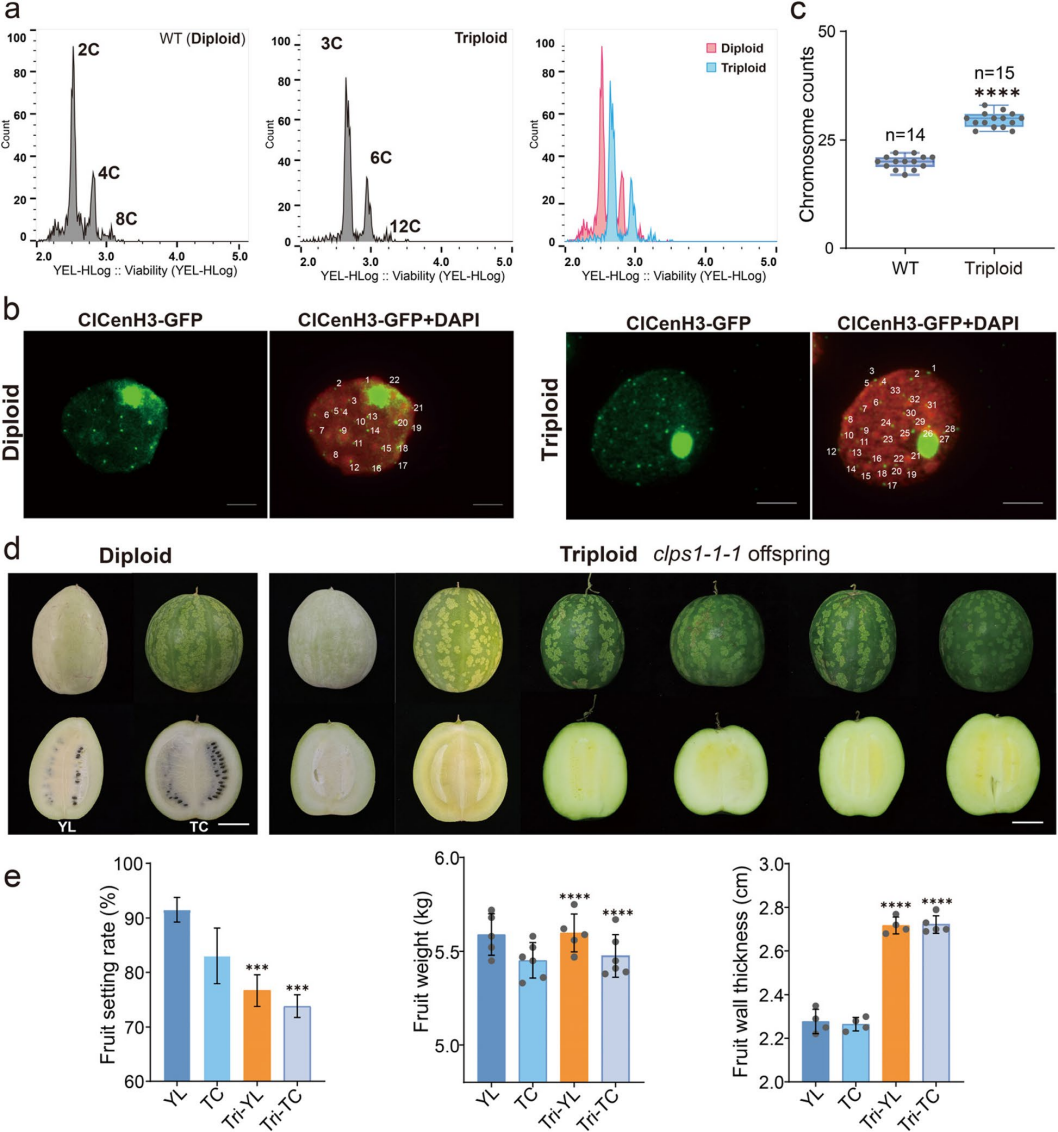
*clps1* plants produce triploid offspring. **a** Comparison of wild-type diploid plants and triploid plants from the offspring of the *clps1* mutant. Scale bars, 10 cm. **b** Immuno-staining of *clps1* triploid offspring plantlets nuclei using a watermelon centromere-specific ClCenH3 antibody. The wildtype serves as a diploid control. DNA was counterstained with DAPI (in red). Centromere signals (in green) indicate the chromosome number. Scale bars, 5 μm. **c** Flow cytometry verification of diploid and triploid plants. **d** Diploid ‘TC’ and triploid watermelon fruits that from F1 progeny of *clps1-1–1* self-crosses and ‘TC’** × ***clps1-1–1* crosses. Scale bars, 5 cm. **e** Comparison of fruit set rate, rind thickness, and individual fruit weight between triploid seedless watermelon and diploid watermelon. Tri-YL and Tri-TC respectively represent triploids in the background of two watermelon materials, YL and TC

Building on the previous findings, we hypothesized that 2n gametes might contribute to the production of triploid offspring. To test this, we measured the ploidy levels of *clps1-1–1* progeny using flow cytometry (Fig. 6a). The self-crossed progeny of *clps1-1–1* plants exhibited both diploid and triploid individuals, with 1.4% of the offspring being triploid (Table 1).

**Table 1:** Ploidy levels of offspring plants from *clps1 - 1–1* and WT parents and their F_1_ hybrids. n = number, 2x = diploid, 3x = triploid, 4x = tetraploid

Mother	Father	Sown(n)	Germinated rate (%)	2** × **(%)	3** × **(%)		4** × **(%)	Aneuploid (%)
WT	WT	120	98.3	100		0	0	0
WT	*clps1 - 1–1*	580	92.5	90.8		1.6	0	1.2
*clps1 - 1–1*	WT	250	94.4	100		0	0	0
*clps1 - 1–1*	*clps1 - 1–1*	518	91.7	91.1		1.4	0	0.9

The triploid status of these plants was further confirmed through immunofluorescence staining using a watermelon centromere-specific ClCenH3 antibody (Fig. 6b,c). Diploid wild-type plants (2n = 22) displayed 22 fluorescence signals, while triploid plants (3n = 33) exhibited 33 fluorescence signals (Fig. 6b,c), providing clear evidence of their triploid status. Notably, no tetraploid plants were detected, consistent with the expectation that the loss of *ClPS1* only affects male meiosis, with female meiosis remaining unaffected.

To further validate this conclusion, we used *clps1-1–1* as the maternal parent and wild-type plants as the paternal parent to determine the ploidy of hybrid offspring. The results revealed that, apart from diploid progeny, no other ploidy levels were observed (Table 1). Additionally, when using the wild-type ‘TC’ as the maternal parent and *clps1-1–1* as the paternal parent, 1.6% of the offspring were identified as triploid (Table 1). These findings strongly suggest that the triploid offspring of *clps1* mutants are exclusively produced through the formation of unreduced male gametes. Notably, the frequency of triploid formation was significantly lower than the proportion of 2n pollen production. To investigate further, we performed pollen germination experiments with the *clps1* mutant (Tushabe and Rosbakh 2021). The results showed no significant difference in the germination rate between 2n and normal pollen (Figure S4). However, we observed some anomalies in the germination of 2n pollen. First, some 2n pollen grains exhibited four apertures, one more than the wild-type (WT) normal pollen. Second, there was variability in the number of pollen tubes formed. While WT haploid pollen typically produces a single pollen tube, some 2n pollen developed two tubes (Figure S4). Recent studies have reported significant morphological and physiological abnormalities in polyploid pollen tubes, which can reduce fertility (Westermann et al. 2024). These germination defects may help explain the low frequency of triploid formation.

Since triploid plants are sterile, they require diploid plants for pollination to set fruit. To confirm this, we cross-pollinated the triploid plants with wild-type pollen and successfully produced seedless triploid watermelon fruits (Fig. 6d). Furthermore, we measured several agronomic traits of these triploid seedless watermelon fruits, including the fruit-setting rate, single-fruit weight, and rind thickness. Compared to wild-type diploid watermelons, the single-fruit weight of triploid watermelons did not show a significant change. However, the rind thickness increased notably, while the fruit-setting rate decreased significantly, both phenotypes being consistent with their ploidy level (Fig. 6e).

## Discussion

Studies in *Arabidopsis thaliana* have shown that 30% of progeny from the *atps1* mutant were triploid, a frequency lower than the diploid pollen production rate in the same mutant (D'Erfurth et al. 2008). In this study, while the *clps1* mutant produced a similar proportion of 2n pollen as the *atps1* mutant, the efficiency of generating triploid offspring was significantly lower compared to *Arabidopsis thaliana*. Therefore, selectively isolating 2n pollen and applying it to hybrid pollination may improve triploid efficiency. Current methods for isolating diploid pollen include staining and size-based sorting, advanced imaging techniques, and density gradient centrifugation, which leverages differences in size and density. Additionally, flow cytometry can identify diploid pollen but may cause damage. These methods are challenging to widely apply in hybrid pollination. Thus, further research is needed to refine these techniques.

Moreover, we speculate that, in addition to the reduced germination rate of 2n pollen, the endosperm balance mechanism may be another critical factor contributing to the low triploid formation rate. The fusion of haploid maternal gametes with diploid paternal gametes results in triploid embryos and tetraploid endosperm (Scott et al. 1998). This imbalance in maternal gene dosage can cause arrested or halted endosperm development, which affects embryo development, reduces seed viability (Köhler et al. 2012), and ultimately leads to the abortion of some triploid seeds. However, triploid embryos in *Arabidopsis* exhibit a greater ability to overcome these limitations (D'Erfurth et al. 2008). In contrast, watermelon embryo development appears to be more sensitive to the ratio of paternal to maternal contributions than *Arabidopsis*. Therefore, this sensitivity may be the primary factor contributing to the inefficiency in producing viable triploid offspring in watermelon.

Conversely, when the maternal genome dosage exceeds that of the paternal genome, endosperm development proceeds normally (Köhler et al. 2012), making the use of 2n female gametes a more effective strategy for triploid production. Studies in *Arabidopsis* and tomato have shown that mutations in the *TAM* and *OSD1* genes affect gamete ploidy, leading to the production of unreduced (2n) gametes (D'Erfurth et al. 2009; Wang et al. 2024). Furthermore, our previous work has demonstrated that mutations in the watermelon *OSD1* gene lead to the doubling of both gametic and somatic ploidy, with triploid individuals observed in the subsequent generation (Pang et al. 2024). These findings provide a solid theoretical foundation for cultivating triploid seedless watermelons. Future research should focus on screening and creating mutant materials that produce 2n egg cells.

**Fig. 7:**
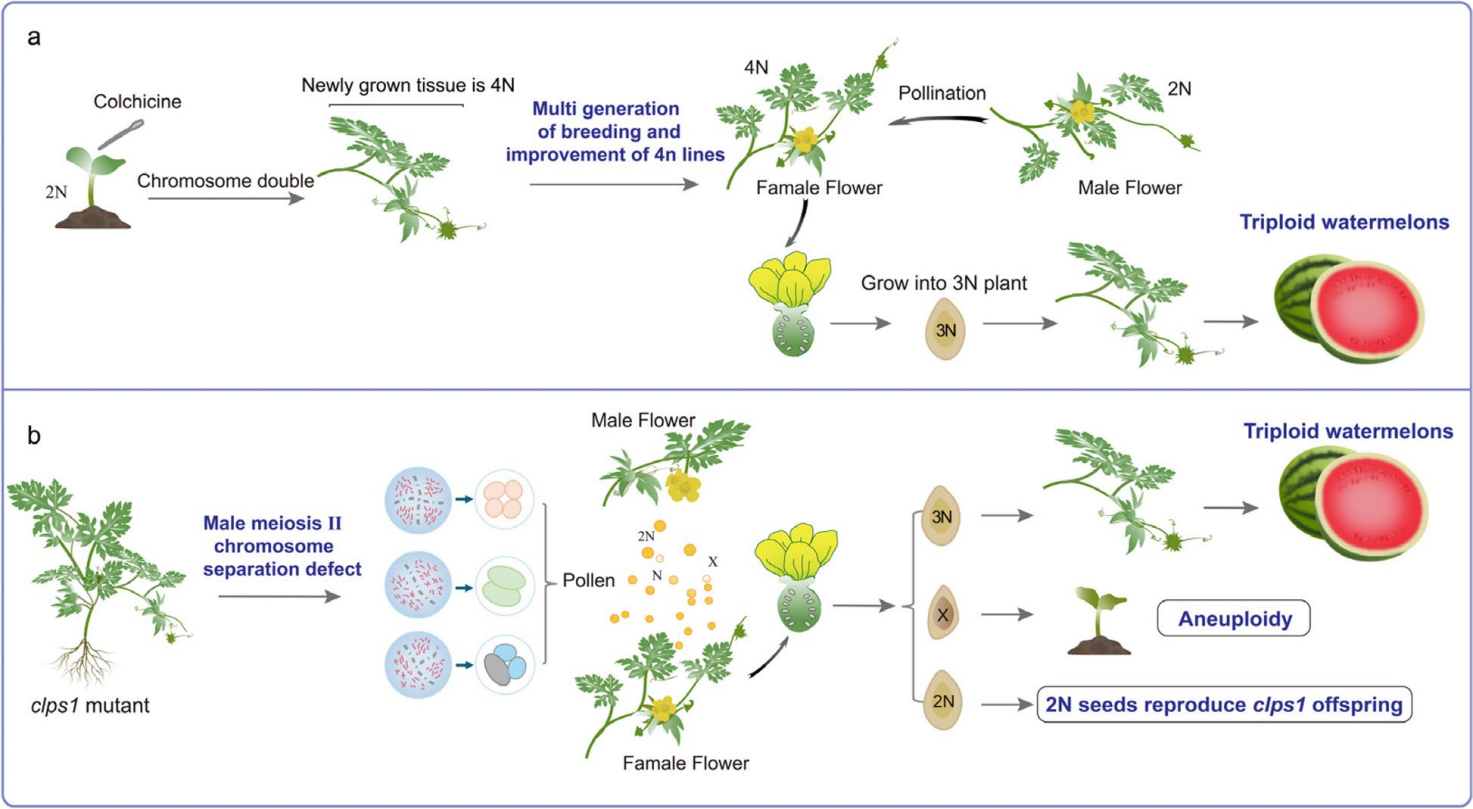
Illustration depicting the cultivation process of triploid seedless watermelon fruits. **a** Traditional breeding method for triploid seedless watermelon. **b** One step creation of triploid seedless watermelon via *clps1* mutants

The core of triploid seedless watermelon breeding lies in the generation of 2n gametes. Traditionally, colchicine treatment is required to obtain tetraploid female parents that produce diploid gametes. Selecting high-quality tetraploid lines and restoring their fertility often takes 5–8 generations, consuming significant manpower and resources. In contrast, the *clps1* mutant can directly generate 40% diploid gametes in a single generation. By hybridizing or self-crossing, this mutant produces triploid seeds in one step (Fig. 7), significantly shortening the breeding cycle and bypassing the complex procedures of traditional methods. Moreover, the *clps1* mutant yields numerous normal diploid seeds, enabling infinite self-propagation (Fig. 7). In summary, to overcome the challenges currently faced in breeding and cultivating seedless watermelons, we have demonstrated that the *ClPS1* gene mutation in watermelon can produce diploid gametes, providing the first direct evidence that the creation of 2n gametes for triploid seedless watermelon breeding is feasible. Notably, we found in the offspring of *clps1* that, in addition to triploids, there were also aneuploid individuals. This is the first report of such plants surviving in watermelon. Although these plants exhibited various developmental disorders and ultimately failed to survive, this discovery provides us with a new avenue of exploration. For example, could crossing *clps1* pollen with cucurbit crops closely related to watermelon help overcome chromosomal imbalance and generate a new species? Relevant experiments are currently underway.

Seedless fruits, prized for their appealing texture and convenience, have gained widespread consumer demand and are expanding from watermelons to other horticultural crops like melons, wax gourds, grapes, and citrus (Pandolfini 2009; Wijesinghe et al. 2020). Although plant growth regulators can produce seedless fruits, their commercial use is often limited due to issues such as fruit deformity, high production costs, and safety concerns (Mesejo et al. 2016; Qian et al. 2018). Our study also included the construction of a phylogenetic tree and the analysis of conserved domains of the PS1 protein across various horticultural crops, including watermelon, melon, cucumber, apple, and grape. The results demonstrated that the *PS1* gene is highly conserved across these species. It is anticipated that, by implementing this strategy, an increasing variety of seedless fruits will be produced in the future.

## Materials and methods

### Plant material

The wild-type watermelon materials used in this experiment were ‘YL’ and ‘TC’, which were obtained and stored in our research group. All the mutants and wild type materials were planted in the experimental field of Northwest A&F University, Yangling.

### Phylogenetic analysis

The putative PS1 homologous protein sequences were obtained from the following websites. JGI Phytozome (https://phytozome-next.jgi.doe.gov/), *Arabidopsis thaliana* (https://www.arabidopsis.org/), C. lanatus (http://cucurbitgenomics.org/). then aligned with ClustalW using default parameters. Artificial modification of the part of the misplaced protein near the central protein sequence. Evolutionary analysis was performed with the neighbor-joining method using the bootstrap method with 1,000 replications by MEGA X (http://www.megasoftware.net/). Protein sequence and alignment in Figure S1.

### RT-qPCR assays

Total RNA was isolated using TRIzol reagent (Invitrogen, Waltham, MA, USA) from watermelon tissues (roots, stems, leaves, and anthers of different developmental stages). Total RNA (2 μg) was treated with DNase buffer to eliminate genomic DNA contamination, and RT reactions were conducted using the Fasting RT Kit (TIANGEN) according to the manufacturer's instruction. The RT product was used as the template for PCR. RT-qPCR was performed with SYBR Green PCR Master Mix (Takara) in a final volume of 10 μl. The relative quantification of gene expression was performed by contrastive CT method (Livak and Schmittgen 2001). Three bioreplicates and three technique repeats PCR analysis were performed for each gene using watermelon Actin gene (gene ID Cla007792) as the internal reference.

### In situ hybridization of male anther development stages

Flower buds fixation, chromosome spreads, and in situ hybridization were performed as previously described with minor modifications (Li et al. 2014; Ren et al. 2018). A 392 bp RNA probe was designed based on the cDNA sequence of *ClPS1*. And DIG RNA Labeling Kit (Roche, Rotkreuz, Switzerland) was used to label the sense and antisense probes of *ClPS1*, according to the manufacturer’s instructions.

Male flower buds were vacuumed in 4% paraformaldehyde and fixed at 4℃ for 24 h. After dehydration, xylene wax was soaked at 42℃, and then the sample was embedded and sliced after the material was cooled naturally. Note that the slide used at this time must be guaranteed to be RNase free. Samples were incubated in prehybridization buffer, 6 × SSC, 0.1% SDS, 50% formamide, 100 μg/ml tRNA at 42℃ for 3 h and DIG-labeled RNA probes were added at 42℃ for overnight hybridization. Incubate in BCIP/NBT solution and finally observe the signal.

### Genetic transformation and genotype analysis in watermelon

Watermelon seeds are first disinfected with 3% sodium hypochlorite and 75% alcohol, then placed on basic MS solid culture medium for seeding for 3 days. Next, watermelon cotyledons are cut into small pieces approximately 1.5 × 1.5 mm in size. Transformation is carried out using *Agrobacterium* strain EHA105 by soaking the watermelon cotyledons in diluted Agrobacterium solution (OD600 = 0.6–0.8) for 15 min. After that, co-cultivation is conducted in darkness for 4 days and then transferred to selection induction medium containing 1.5 mg/L 6-BA and 200 mg/L Timentin. The culture medium is changed every 10 days.

Genomic DNA was extracted from each transgenic leaf tissue. Specific primers were utilized for PCR amplification of the target genomic regions (Table S3), and the resulting PCR products underwent Sanger sequencing. Furthermore, a subset of high-quality sequencing data from the PCR products was selected for deep sequencing on the Hi-Tom (High-Throughput Mutation) platform. The obtained sequencing data was then uploaded to the Hi-TOM online analysis website (Hi-TOM: http://www.hi-tom.net/hi-tom/index-EN.php) to determine the specific types of editing at each locus for every plant, enabling comprehensive analysis of the sequencing data.

### Alexander staining of pollen

Male flowers of both WT and *clps1* mutant that bloomed on the same day were collected. The pollen was gently shaken onto a microscope slide, followed by the addition of 60 μl of Alexander staining solution. After 30 min of staining, the viability of pollen was observed and captured using a microscope (Axio Imager M2; Zeiss).

### Flow cytometric assay

The sample was prepared according to the described (Borges et al. 2012; Chen et al. 2023) and minor changes were made. Fresh leaves of each material to be measured were placed in Cell lysate LB01 buffer with the same growth state as WT plants. The leaves were continuously chopped vertically with a sharp blade. And nuclear solution was collected through a 35 μm cell filter, stained with 50 μg/ml PI (Propidium Iodide), and analyzed on Muse Cell Analyzer (Luminex). Typically, 2000 nuclei were analyzed per sample. The DNA content of the mutant was calculated according to the location of 2 C or 4 C peaks of the wild type and mutant.

### Cytological analysis of male meiosis

Flower buds of different sizes were taken according to the male flower reproductive calendar of watermelon, fixed in Carnoy Fluid, and stored at − 20℃ as possible. we performed this procedure as previously reported (Ross et al. 1996; Tian et al. 2021).The flower was peeled and washed three times in 0.01 M citrate buffer (4 ml of 0.1 M citric acid, 6 ml of 0.1 M sodium citrate, pH 4.5) for 5 min then treated with 2% cellulase (Sigma-Aldrich) and 0.25% pectinase (Sigma-Aldrich) in citrate buffer for 3 h in a moist chamber at 37℃. The anthers were crushed with tweezers, each slide was added with 10 μl 60% acetic acid solution, covered with the cover slide, pressed vertically with thumb, frozen at − 80℃ for 10 min, removed the cover slide, dried at room temperature, and stained with DAPI.

### Immunofluorescence assay

Specific antibody was designed according to the protein sequence of watermelon ClCenH3, and the antibody was synthesized by the company. We performed this procedure as previously reported with minor modifications (Liu et al. 2024). The cell suspension and sucrose solution were dropped 1:1 on a slide and dried overnight at room temperature. Washed twice with 1 × PBS buffer, added 100 μl 1 st antibody solution (2% BSA in 1 × PBS with 0.1% Tritonex- 100:1 st antibody = 200:1). The slides were incubated overnight in a wet box at 4℃. Washed twice with 1 × PBS buffer. 100 μl of 2nd antibody solution was dropped onto the slide, then left at high humidity at 37℃ for 1 h. Dehydrated with 70%, 90% alcohol and dried the slides at room temperature. Samples were stained with DAPI.

## Supplementary Information

Supplementary Material 1.

## Data Availability

The paper presents all the data generated in this study. Additional data related to this research can be requested from the authors.

## Abbreviations

AtPS1: Arabidopsis thaliana Parallel Spindles1
ClPS1: Citrullus lanatus Parallel Spindles1
OSD1/GIG1: Omission of Second Division/GIGAS Cell 1
TAM(CYCA2;1): Tardy Asynchronous Meiosis
SMC5/6: Structural Maintenance of Chromosome 5/6
AM1: AMEIOTIC1
FDR: First Division Restitution
SDR: Second Division Restitution
FHA Domin: ForkHead Associated Domin
PIN Domin: PilT N-terminus Domin
ClCenH3: Citrullus lanatus Centromere-specific Histones 3
DIG-labeled: Digoxigenin labeled
