# Supplementary material for: ClPS1 gene-mediated manipulation of 2n pollen formation enables the creation of triploid seedless watermelon
Source: Mol Hortic. 2025 Sep 2;5:48. doi: 10.1186/s43897-025-00170-2 (PMC12403409; doi:10.1186/s43897-025-00170-2)
Supplement: Supplementary file 1 — Supplementary Material 1. [file 43897_2025_170_MOESM1_ESM.docx]

**Supplementary figures, tables for**

***ClPS1* Gene-Mediated Manipulation of 2n Pollen Formation Enables the Creation of Triploid Seedless Watermelon**
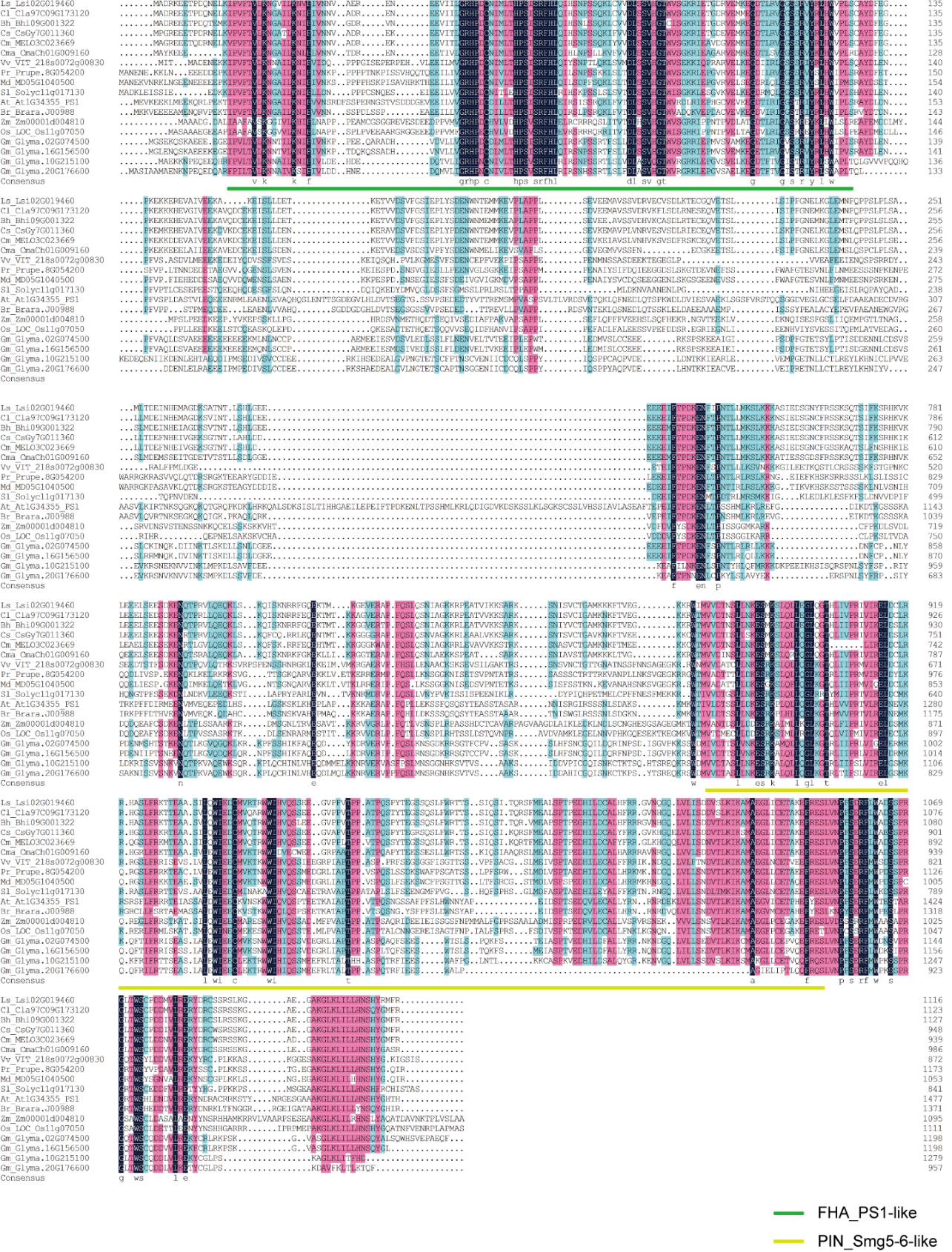


**Figure S1.** **Alignment of PSI protein sequences across multiple species, revealing the two most conserved segments.** Subsequent analysis identified these fragments as belonging to the FHA and PIN families, respectively. Prior to each protein identifier, a two or three letter code denoting the species origin was provided. At, *Arabidopsis thaliana*; Br, *Brassica rapa*; Bh, *Benincasa hispida*; Cs, *Cucumis sativus*; Cm, *Cucumis melo*; Cma,*Cucurbita maxima*; Cl,*Citrullus lanatus*; Gm, *Glycine max*; Ls,*Lagenaria siceraria*; Md, *Malus domestica*; Os, *Oryza sativa*; Pr, *Prunus persica*; Sl, *Solanum lycopersicum*; Vv, *Vitis vinifera* ; Zm, *Zea mays*.


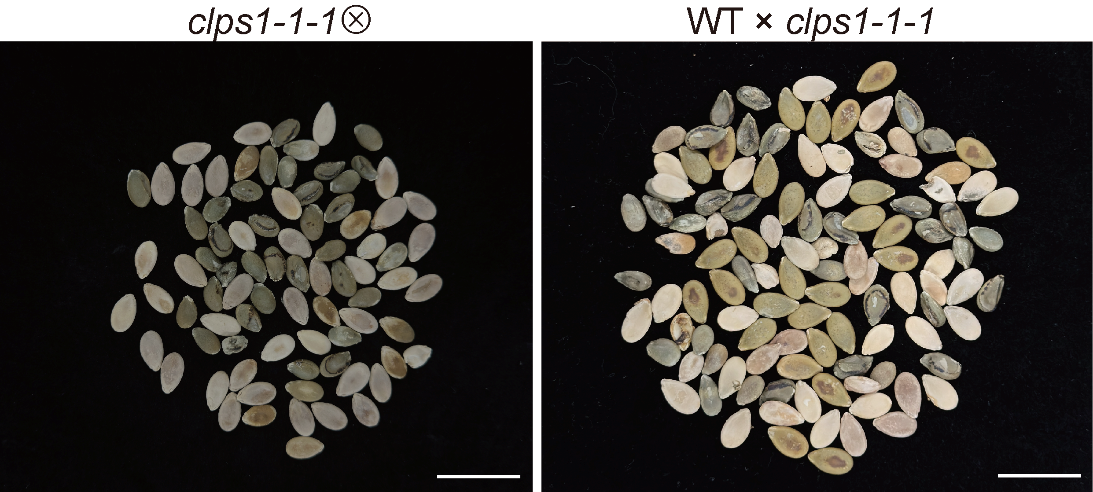


**Figure S2.** **Abnormal seeds for *clps1* mutant.** Abnormal seeds included lighter seed color and abnormal shape and size. Scale bars, 5 cm


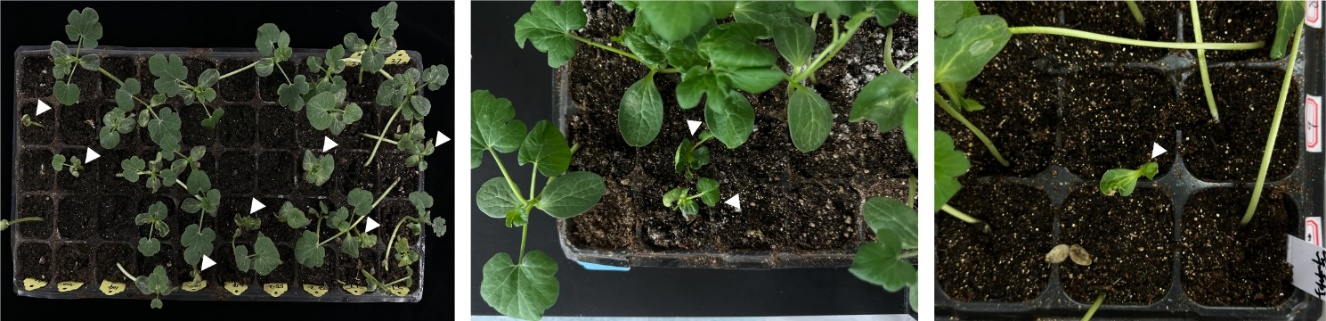


**Figure S3.** **Plant morphology of offspring of** ***clps1* abnormal seeds.** The white arrow marks the aneuploid plants, which show slow development, short plants, and irregular leaves.


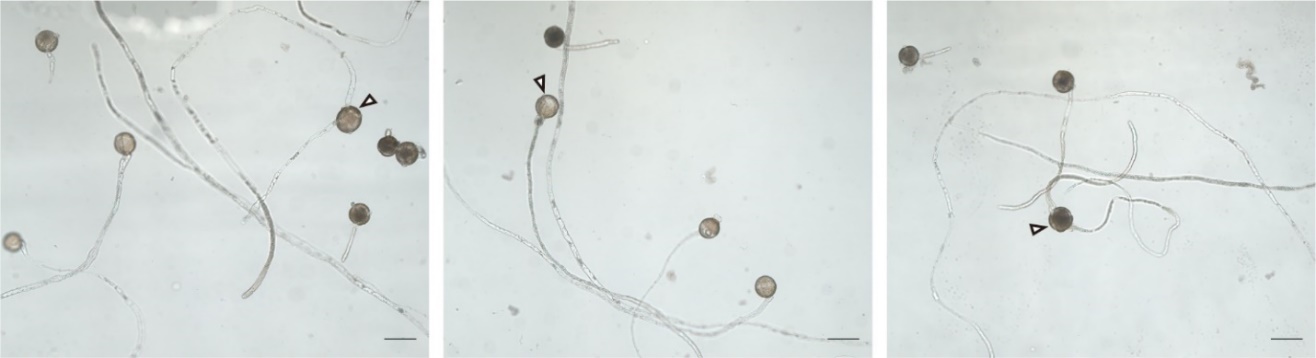


**Figure S4**. **Pollen germination of *clps1* mutants.** The pollen was evenly dithered onto germination medium (0.045% boric acid, 10% sucrose and 5% PEG3350) and placed at 28°C for 1 to 2 hours under moisturizing conditions. Larger pollen is indicated by arrows. Some larger pollen grains of the *clps1* mutant produced more than one pollen tube compared with haploid pollen. Scale bars, 100 μm.

**Supplemental Table 1. Editing events in mutants analyzed by HI-TOM sequencing.**

| **Mutant name** | **Mutant sites** | **sgRNA1 mutation** | **sgRNA2 mutation** | **CDS length(bp)** | **Protein length(aa)** |
| --- | --- | --- | --- | --- | --- |
| WT *ClPS1* | - | TCTTCACCGTTCTCAAGAA**CGG** | **CCT**TGGACGACACCCAGATTGC | 3372 | 1124 |
| *clps1-1* | WT 43.6%  -4bp 42.8% | TCTTCACCGTTCTCAAGAA**CGG**  TCTTCACCGTTC----GAA**CGG (-4bp)** | **CCT**TGGACGACACCCAGATTGC | 3372/96 | 1124/32 |
| *clps1-3* | -3bp 90.5% | TCTTCACCGTTC---AGAA**CGG (-3bp)** | **CCT**TGGACGACACCCAGATTGC | 3369 | 1123 |
| *clps1-4* | +1bp 40.7%  -3bp 43.5% | TCTTCACCGTTCTCAA**A**GAA**CGG(+1bp)**  TCTTCACCGTTC---AGAA**CGG (-3bp)** | **CCT**TGGACGACACCCAGATTGC | 192/3369 | 64/1123 |
| *clps1-5* | -3bp 44.7%  WT 43.6% | TCTTCACCGTTC---AGAA**CGG (-3bp)**  TCTTCACCGTTCTCAAGAA**CGG** | **CCT**TGGACGACACCCAGATTGC | 3369/3372 | 1123/1124 |
| *clps1-6* | -3bp 71.2%  -2bp 22.2% | TCTTCACCGTTC---AGAA**CGG (-3bp)**  TCTTCACCGTTCTC--GAA**CGG (-2bp)** | **CCT**TGGACGACACCCAGATTGC | 3369/189 | 1124/63 |
| *clps1-7* | -18bp 29.4%  -2bp 22.8%  +1bp 22.1%  -4bp 17.8% | TCTTCACCG-----------------**(-18bp)**  TCTTCACCGTTCTC--GAA**CGG (-2bp)**  TCTTCACCGTTCTCAA**A**GAA**CGG(+1bp)**  TCTTCACCGTTC----GAA**CGG (-4bp)** | **CCT**TGGACGACACCCAGATTGC | 3354  189  192  96 | 1118  63  64  32 |
| *clps1-8* | +1bp 45.7%  WT 45.2% | TCTTCACCGTTCTCAA**A**GAA**CGG(+1bp)**  TCTTCACCGTTCTCAAGAA**CGG** | **CCT**TGGACGACACCCAGATTGC | 192/3372 | 64/1124 |

Note: PAM sequences (CCN or NGG) were highlighted by red colors.( The mosaics were not listed.) HI-TOM: http://www.hi-tom.net/hi-tom/index-CH.php

| **Potential off-target site** | **Sequence** | **MMs** | **Locus** | **Gene ID** | **Region** |
| --- | --- | --- | --- | --- | --- |
| *clps1*-gRNA1 | TCTTCACCGTTCTCAAGAA**CGG** |  |  |  |  |
| *clps1*-off target1-1 | **A**CTTC**CT**C**A**TTCTCAAGAA**CGG** | 4MMs | Chr6:+18593580 |  | Intergenic |
| *clps1*-off target1-2 | **GA**TTCACC**T**TTCTCAAG**T**A**CGG** | 4MMs | Chr10:-12654824 |  | Intergenic |
| *clps1*-off target1-3 | T**T**TTCA**T**C**T**TTCTC**C**AGAA**TGG** | 4MMs | Chr3:-8380088 | Cla019671 | cds |
| *clps1*-off target1-4 | TCTTCA**G**CGTTCT**GC**AGAA**AGG** | 3MMs | Chr8:+19210378 | Cla021972 | cds |
| *clps1*-gRNA2 | GCAATCTGGGTGTCGTCCA**AGG** |  |  |  |  |
| *clps1*-off target2-1 | GC**C**AT**T**TGGG**G**GTCG**A**CCA**TGG** | 4MMs | Chr9:+27027955 |  | Intergenic |
| *clps1*-off target2-2 | GCAATCT**CA**GT**A**TC**T**TCCA**AAG** | 4MMs | Chr8:+19069850 | Cla021960 | cds |
| *clps1*-off target2-3 | GCAA**A**CTGGGTGT**T**G**AA**CA**CGG** | 4MMs | Chr10:+20899505 | Cla017014 | cds |

**Supplemental Table 2. Sanger sequencing was used to analyze the mutations of the off-target site.**


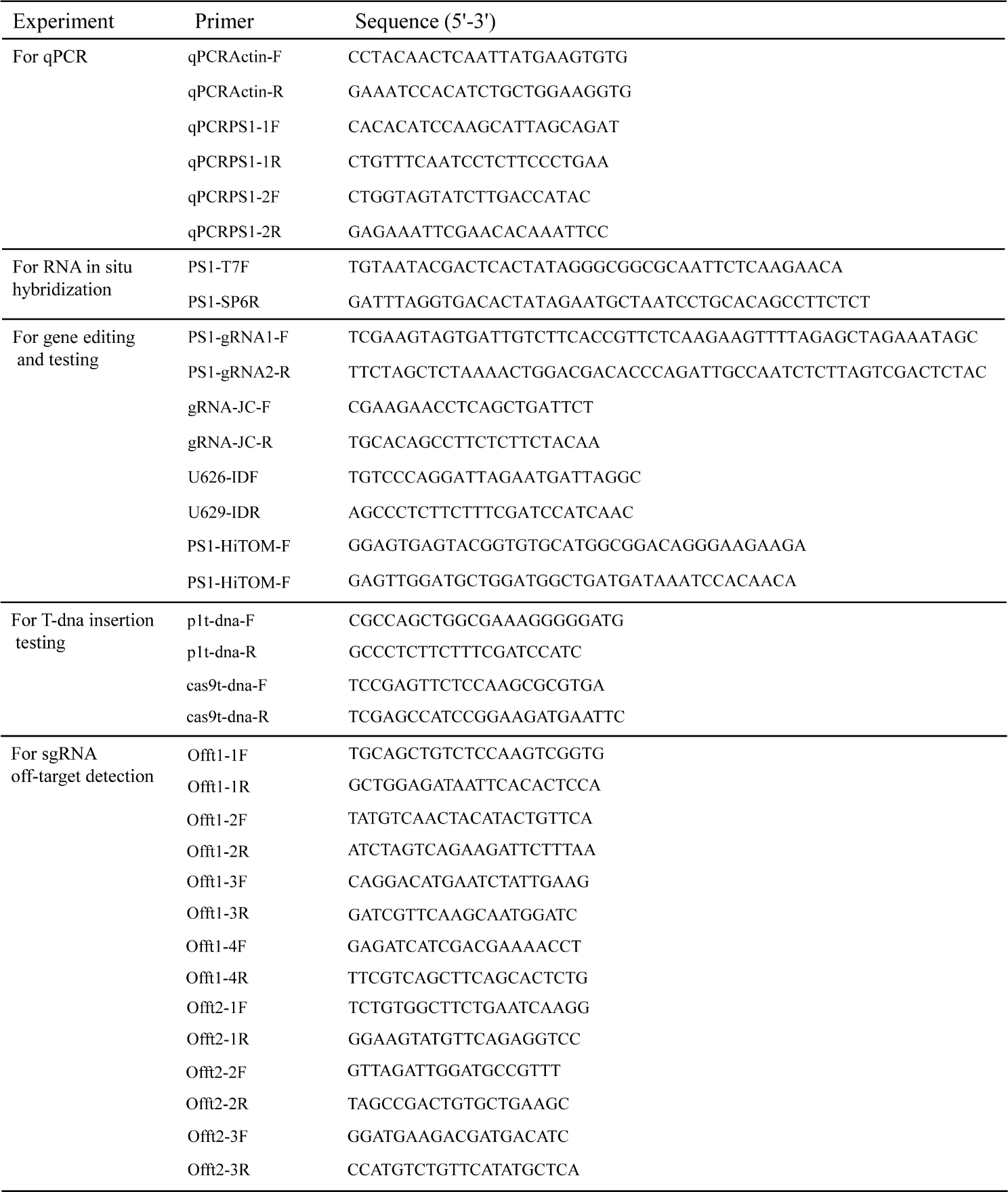
**Supplemental Table 3. Primers used in this study**
